# Donor Recipient Chimeric Cells Induce Chimerism and Extend Survival of Vascularized Composite Allografts

**DOI:** 10.1007/s00005-021-00614-9

**Published:** 2021-05-10

**Authors:** Joanna Cwykiel, Arkadiusz Jundzill, Aleksandra Klimczak, Maria Madajka-Niemeyer, Maria Siemionow

**Affiliations:** 1 Department of Orthopaedics, University of Illinois At Chicago, Molecular Biology Research Building, 900 S. Ashland Ave. Room# 3356, Chicago, IL 60607 USA; 2 Department of Plastic Surgery, Cleveland Clinic, Cleveland, OH USA; 3 Chair of Urology, Department of Regenerative Medicine, Nicolaus Copernicus University in Torun, Ludwik Rydygier Medical College in Bydgoszcz, Bydgoszcz, Poland; 4 Department of Plastic, Reconstructive and Aesthetic Surgery, Collegium Medicum in Bydgoszcz, Nicolaus Copernicus University in Toruń, Bydgoszcz, Poland; 5 Hirszfeld Institute of Immunology and Experimental Therapy, Polish Academy of Sciences, Wroclaw, Poland; 6 Department of Surgery, Poznan University of Medical Sciences, Poznan, Poland

**Keywords:** Donor recipient chimeric cells, Vascularized composite allotransplantation, Cell fusion, Groin flap

## Abstract

This study evaluated the efficacy of donor recipient chimeric cell (DRCC) therapy created by fusion of donor and recipient derived bone marrow cells (BMC) in chimerism and tolerance induction in a rat vascularized composite allograft (VCA) model. Twenty-four VCA (groin flaps) from MHC-mismatched ACI (RT1^a^) donors were transplanted to Lewis (RT1^l^) recipients. Rats were randomly divided into (*n* = 6/group): Group 1—untreated controls, Groups 2—7-day immunosuppression controls, Group 3—DRCC, and Group 4—DRCC with 7-day anti-αβTCR monoclonal antibody and cyclosporine A protocol. DRCC created by polyethylene glycol-mediated fusion of ACI and Lewis BMC were cultured and transplanted (2–4 × 10^6^) to VCA recipients via intraosseous delivery route. Flow cytometry assessed peripheral blood chimerism while fluorescent microscopy and PCR tested the presence of DRCC in the recipient’s blood, bone marrow (BM), and lymphoid organs at the study endpoint (VCA rejection). No complications were observed after DRCC intraosseous delivery. Group 4 presented the longest average VCA survival (79.3 ± 30.9 days) followed by Group 2 (53.3 ± 13.6 days), Group 3 (18 ± 7.5 days), and Group 1 (8.5 ± 1 days). The highest chimerism level was detected in Group 4 (57.9 ± 6.2%) at day 7 post-transplant. The chimerism declined at day 21 post-transplant and remained at 10% level during the entire follow-up period. Single dose of DRCC therapy induced long-term multilineage chimerism and extended VCA survival. DRCC introduces a novel concept of customized donor-recipient cell-based therapy supporting solid organ and VCA transplants.

## Introduction

The continued effort to introduce new solutions into the field of transplantation is driven by the need to limit the use of anti-rejection protocols which, although necessary, are associated with severe co-morbidities and significant shortening of transplant recipient’s lifespan.

Cell-based therapies were proposed as a promising supportive treatment due to observed direct and indirect involvement of cells of hematopoietic and mesenchymal origin in the alteration of the local and systemic immune response of the transplant recipients (Siemionow et al. 2012). Research effort focused on bone marrow transplantation (BMT) and bone marrow cell (BMC) based therapies provided encouraging results supporting the hypothesis of the importance of long-term chimerism in transplant tolerance induction (Leventhal and Ildstad 2018; Mathes et al. 2014; Scandling et al. 2008; Siemionow et al. 2002a, b, 2003, 2008). The experimental in vitro studies of BMC and clinical BMT studies reported the occurrence of cells presenting phenotype and/or genotype make-up specific for both the transplant donor and recipient and suggested contribution of the donor/recipient cells to the processes such as regeneration and immune response (Alvarez-Dolado et al. 2003; Camargo et al. 2004; Johansson et al. 2008; LaBarge and Blau 2002; Lluis and Cosma 2010; Nygren et al. 2004; Powell et al. 2011; Rizvi et al. 2006; Sanges et al. 2013; Vassilopoulos et al. 2003; Wang et al. 2003; Weimann et al. 2003; Willenbring et al. 2004). These cells are a product of either cell fusion, an ubiquitous process of asexual merging of two or more parental cells (Dittmar and Zanker 2015; Zito et al. 2016), or trogocytosis, a process of transfer of cell membrane fragments with associated proteins between cells (Ahmed et al. 2008). Our group, following the detection of donor/recipient cells in a rat BMT recipient, developed a protocol to create donor-recipient chimeric cells (DRCC) in vivo and tested the effect of DRCC’s in a fully MHC-mismatched rat vascularized composite allotransplantation (VCA; hemiface) model (Hivelin et al. 2016). Application of DRCC as a supportive therapy under a short-term immunosuppression (IS) protocol of anti-αβTCR monoclonal antibody and cyclosporine A (anti-αβTCR/CsA) was associated with prolonged VCA survival (Hivelin et al. 2016). The improvement of VCA survival time could have been caused by the combination of multi-lineage chimerism induction and/or immunomodulatory properties of in vivo created DRCC (Hivelin et al. 2016).

Based on these encouraging results, we have created a novel clinically feasible DRCC therapy using an ex vivo polyethylene glycol (PEG)-mediated fusion of BMC isolated from transplant donor and recipient (Cwykiel et al. 2021). Our study confirmed the chimeric phenotype and genotype make-up of DRCC using flow cytometry, confocal microscopy, and PCR. In addition, we showed that the applied ex vivo cell fusion procedure was not genotoxic and did not change the expression of the hematopoietic markers, proliferation rate or differentiation potential of DRCC compared to BMC controls. In vitro evaluation indicated pro-tolerogenic profile of DRCC presented by decreased immunogenicity and secretion of pro-tolerogenic IL-10 and TGFβ1 cytokines (Cwykiel et al. 2021).

This study focused on the assessment of immunomodulatory effect of DRCC therapy in vivo to support tolerance induction in a rat VCA (groin flap) model containing highly immunogenic skin component. In addition, we evaluated the potential of DRCC for chimerism induction and DRCC’s migration pattern in vivo at the experimental endpoint.

## Materials and Methods

### Experimental Design

Cleveland Clinic’s Institutional Animal Care and Use Committee (IACUC, Cleveland, OH), accredited by the American Association for the Accreditation of Laboratory Animal Care (AAALAC, #2012-0841), approved this study. All animals received humane care in compliance with the “Principles of Laboratory Animal Care” formulated by the National Society for Medical Research and the “Guide for the Care and Use of Laboratory Animal Resources” (2011) published by the US National Institutes of Health.

**Fig. 1:**
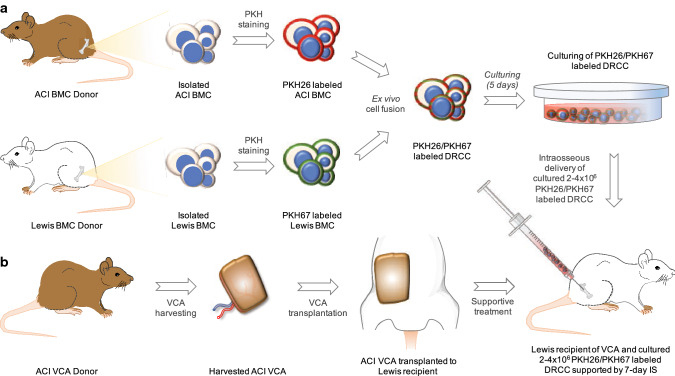
Diagram of the experimental study design. **a** Creation of donor recipient chimeric cells (DRCC) therapy via ex vivo cell fusion. From left: bone marrow cells (BMC) were isolated from ACI (RT1^a^) and Lewis (RT1^l^) donor rats and labeled with PKH26 (ACI BMC) or PKH67 (Lewis BMC). PKH labeled BMC of ACI and Lewis origin were mixed, and PEG-mediated fusion procedure was performed. Double PKH26/PKH67 labeled DRCC were sorted and cultured in “enriched” StemSpan® SFEM medium. Following 5-day culture, DRCC were applied as a supportive therapy for vascularized composite allograft (VCA) recipients. **b** VCA (groin flap) transplantation and application of DRCC therapy. From left: VCA was harvested from ACI (RT1^a^) donor rat and transplanted to the groin region of fully MHC-mismatched Lewis (RT1^l^) recipient. Following transplantation, VCA recipients received intraosseous injection of cultured 2–4 × 10^6^ DRCC and a 7-day immunosuppression (IS) protocol of anti-αβTCR monoclonal antibody and CsA

A total of 24 vascularized skin allografts (VSA; groin flaps) have been transplanted between 7 and 10 weeks old male ACI (August Copenhagen Irish, RT1^a^) donors and Lewis (RT1^1^) recipients (weight 250–300 g; Envigo, USA). The VCA Lewis recipients were randomly divided into four groups (*n* = 6/group): Group 1—did not receive any treatment; Group 2—was supported by 7-day anti-αβTCR/CsA immunosuppression (IS) protocol; Group 3—was supported with DRCC (2–4 × 10^6^ cells) therapy delivered via intraosseous injection to recipient’s femur; and Group 4—was supported with intraosseous delivery of DRCC (2–4 × 10^6^ cells) therapy and 7-day anti-αβTCR/CsA IS protocol. The IS protocol included intraperitoneal injection of anti-αβTCR monoclonal antibody (250 µg/day, clone R73; BD Pharmingen, USA) and subcutaneous injection of CsA (16 mg/kg/day; Bedford Laboratories, USA). The experimental design is presented in detail in Fig. 1.

### Creation of DRCC Therapy via Ex Vivo Cell Fusion

Forty-eight male 7–8 weeks old ACI (RT1^a^) and 48 Lewis (RT1^l^) rats served as donors for the creation of DRCC therapy (Envigo, USA). BMC were isolated in a sterile manner, as previously described (Cwykiel et al. 2021; Hivelin et al. 2016; Siemionow et al. 2016), from femurs and tibias using flashing technique. Harvested cells were filtered using 40 µm strainer, purified by Histopaque 1084 (Millipore–Sigma, USA) and counted with 0.4% Trypan Blue (Thermo Fisher Scientific, USA). Next, BMC from each donor strain were stained either with PKH26 (ACI) or PKH67 (Lewis) fluorescent dyes (Millipore–Sigma, USA) for 3 min as previously reported (Cwykiel et al. 2021; Siemionow et al. 2016, 2018a; b). Following staining, PKH26-ACI BMC and PKH67-Lewis BMC were mixed and re-suspended in RPMI 1640 medium without fetal bovine serum (FBS). Fusion was performed as previously reported (Cwykiel et al. 2021; Siemionow et al. 2018a, b) using 50% w/v polyethylene glycol 4000 (EMD, USA) and 16% dimethyl sulfoxide (DMSO). PKH26/PKH67 labeled DRCC were selected using Special Order BD FACS Aria II. Purity and viability of DRCC (1 × 10^5^ cells, *n* = 3) were assessed using LSRFortessa cytometer (BD, Franklin Lakes, NJ, USA). The chimeric state of DRCC was confirmed as previously reported by confocal microscopy and flow cytometry (Cwykiel et al. 2021; Siemionow et al. 2018a, b). Next, sorted DRCC were cultured for 5 days in “enriched” StemSpan® SFEM medium containing 10% FBS (Thermo Fisher Scientific), 1 × antibiotics/antimycotic solution (Millipore–Sigma) and cytokine mix (Zhang and Lodish 2005) containing: recombinant human acidic fibroblast growth factor (10 ng/mL), recombinant mouse stem cell factor (10 ng/mL), recombinant mouse thrombopoietin (20 ng/mL), recombinant mouse insulin growth factor-II (20 ng/mL; R&D Systems, USA), and heparin (10 µg/mL; Millipore–Sigma, USA).

### Vascularized Composite Allograft (Groin Flap) Transplantation

The technical details of the groin flap transplantation procedure were previously published (Demir et al. 2005; Siemionow et al. 2016). Briefly, the right groin and leg area of donor and recipient rats anesthetized with subcutaneous injection of ketamine (30 mg/kg), acepromazine (6 mg/kg), and xylazine (1 mg/kg), were shaved, sterilized, and draped in a sterile fashion. Surgical marker was used to outline the area of allograft including the xiphoid and pubis midline, the inguinal ligament inferiorly, costal arch superiorly, and axillary line laterally. Transplantation procedures were performed under 40 × operating microscope magnification.

### VCA Harvest

The 3 × 3 cm groin flap was raised exposing the pedicle, and a retractor was placed in the caudal aspect of the VCA. The lateral femoral circumflex artery and vein, and any additional femoral branches to adjacent muscles were ligated using 10–0 nylon monofilament suture. A pair of straight microsurgical scissors was used to cut the isolated femoral artery and vein and trim a cuff of adventitia close to the vascular section. Heparinized normal saline (10 IU/ml) was used to flush the lumens of femoral vessels while Addison’s forceps were applied to transfer the VCA. The groin flap was kept in moist gauze pre-transplantation.

### VCA Recipient Preparation

The same method of VCA dissection as for ACI donor was also applied for the Lewis recipient. The transected femoral vessels were flushed with heparinized normal saline (10 IU/ml) and the recipient’s graft (3 × 3 cm) was removed creating the defect required for transplantation of the ACI donor VCA.

### Transplantation Procedure

Following VCA transfer, end-to-end anastomosis between the ACI donor and Lewis recipient femoral arteries and veins were performed under operating microscope (magnification × 40) with 10–0 nylon monofilament sutures using standard microsurgical techniques. Warm ischemia time upon the grafts’ revascularization was limited to approximately 55 min in each case. The patency of anastomoses was confirmed, and the vessels were inspected for signs of hemorrhage, thrombosis or excessive traction. After restoration of perfusion subcutaneous interrupted sutures (5–0 Vicryl, Ethicon) were used to close the skin (Demir et al. 2005). VCA recipients in Groups 2 and 4 received subcutaneous injections of CsA (16 mg/kg/day) and intraperitoneal injection of anti-αβT cell receptor (TCR) monoclonal antibody (250 µg/day) for 7 days with the first injections at the day of VCA transplantation. Buprenorphine (0.075 mg/kg) was administered twice daily for 3 days after the surgery as analgesic. Following the completion of VCA transplantation, the cultured DRCC therapy has been delivered to the VCA recipient’s femoral bone as previously reported (Hivelin et al. 2016; Klimczak et al. 2007; Siemionow et al. 2005a).

### Gross Examination of Transplanted VCA

During the post-transplant period, the animals were evaluated daily for the presence of any signs of pain or stress, while the VCA were monitored for any clinical signs of rejection, such as erythema, hair loss, edema, epidermolysis, ulceration, graft shrinkage, and mummification. The necrosis of 80% of the flap was considered as a rejection and experimental endpoint (Demir et al. 2005; Hivelin et al. 2016; Lei et al. 2019; Siemionow et al. 2016).

### Evaluation of Peripheral Blood Chimerism in the VCA Transplant Recipients by Flow Cytometry

Blood samples were harvested from VCA recipients at days 7, 21, 35, 63, and 100 post-transplantation. Red blood cells were removed form blood samples using 0.83% NH_4_CI/Tris (Sigma–Aldrich, USA) solution (Klimczak et al. 2007), and isolated cells were washed twice with Dulbecco’s phosphate buffered saline (D-PBS). Further, cells were resuspended in a cell staining buffer solution containing 1% BSA and 0.05% sodium azide in 1 × PBS and incubated with fluorescein isothiocyanate (FITC)- and phycoerythrin (PE)-labeled monoclonal antibodies or isotype controls for 30 min on ice. The following monoclonal antibodies were used for staining: FITC-conjugated: mouse anti-rat CD3 (BD554832), rat anti-rat RT1^ab^, (BD550981), mouse anti-rat CD4 (BD554837), and mouse anti-rat αβ-TCR (BD554913), as well as PE-conjugated PE mouse anti-rat CD45RA, (BD554884), mouse anti-rat CD4 (BD554838), mouse anti-rat CD8 (BD554857), mouse anti-rat γδ-TCR (BD551802), and mouse anti-rat CD25 (BD554866). Next, cells were washed twice with cell staining buffer and fixed with 1% neutral buffered formalin in 1 × PBS overnight. Labeled cells were analyzed on a FACSScan (Coulter, EPICS Elite-ESP) and BD LSRFortessa™ (BD Biosciences, USA) flow cytometers. Ten thousand cells were acquired for each tested sample.

### Detection of DRCC Migration In Vivo by Polymerase Chain Reaction and Confocal Microscopy

At the experimental endpoint, the VCA recipients were humanly euthanized by buprenorphine overdose, and blood, bone marrow (BM) from injected bone and lymphoid tissues (thymus, spleen, and lymph node) samples were harvested.

Microscopy analysis: Tissue samples were placed in a Tissue-Tek Cryomold and covered in Tissue-Tek® O.C.T. (Sakura Finetek USA Inc., USA) and flash frozen. Samples were sectioned using Microm HM 525 cryostat (Thermo Fisher Scientific, USA). Sections (5 μm) were fixed in 4% paraformaldehyde for 10 min and then rinsed in Tris buffered saline (Dako, Germany). Slides mounted in Vectashield mounting medium with DAPI (Vector Laboratories, USA) were analyzed using fluorescence microscopes (Leica, Germany). The presence of DRCC within the tissue was assessed based on double PKH26/PKH67 labeling. For analysis of peripheral blood and BM, cells were isolated as described above. Next, blood (100 µl) and BM (2 ml) were lysed using 20 ml of ACK buffer (Thermo Fisher Scientific, USA) for 4 min at room temperature, centrifuged at 300 × g for 5 min and washed with 1 × D-PBS two times. Next, the sample was fixed with 4% paraformaldehyde for 15 min and washed again with 1 × D-PBS. Cells were suspended in 50 µl of 1 × D-PBS and 10 µl was applied on a base slide (Fisherbrand™ Superfrost™ Plus Microscope Slides, USA) together with 1 µl of DAPI solution (300 nM; Thermo Fisher Scientific) and cover with coverglass (Thermo Fisher Scientific, USA). Slides were assessed within 30 min after preparation using Leica TCS-SP upright confocal microscope.

Polymerase chain reaction (PCR) analysis: The samples of peripheral blood (100 µl), BM (1 × 10^6^ cells) as well as thymus, lymph nodes, and spleen were harvested at the experimental endpoint (VCA rejection). DNA was isolated using DNeasy Blood & Tissue Kit (QIAGEN, Germany) according to the manufacturer’s instructions. PCR was performed as previously described (Siemionow et al. 2016).

### Statistical Analysis

All results are presented as mean ± standard deviation (SD). A log-rank test was used to assess the overall difference in freedom of rejection among experimental groups. For chimerism evaluation: the normal distribution of the sample was confirmed using Anderson–Darling test. The GLM analysis assessed the mean differences among experimental groups and was followed by post hoc Bonferroni test compared the trend over time were assessed by examining the interaction between group and time. The comparison of groups’ means was performed for four groups with a Bonferroni-adjusted significance level for multiple comparisons. Minitab 2017 software (OriginLab Corp. USA) was used to perform statistical analysis. *p < 0.05, **p < 0.01, ***p < 0.001.

## Results

### Application of DRCC Supportive Therapy is Associated with Significantly Prolonged Survival of Fully MHC-Mismatched VCA

**Fig. 2:**
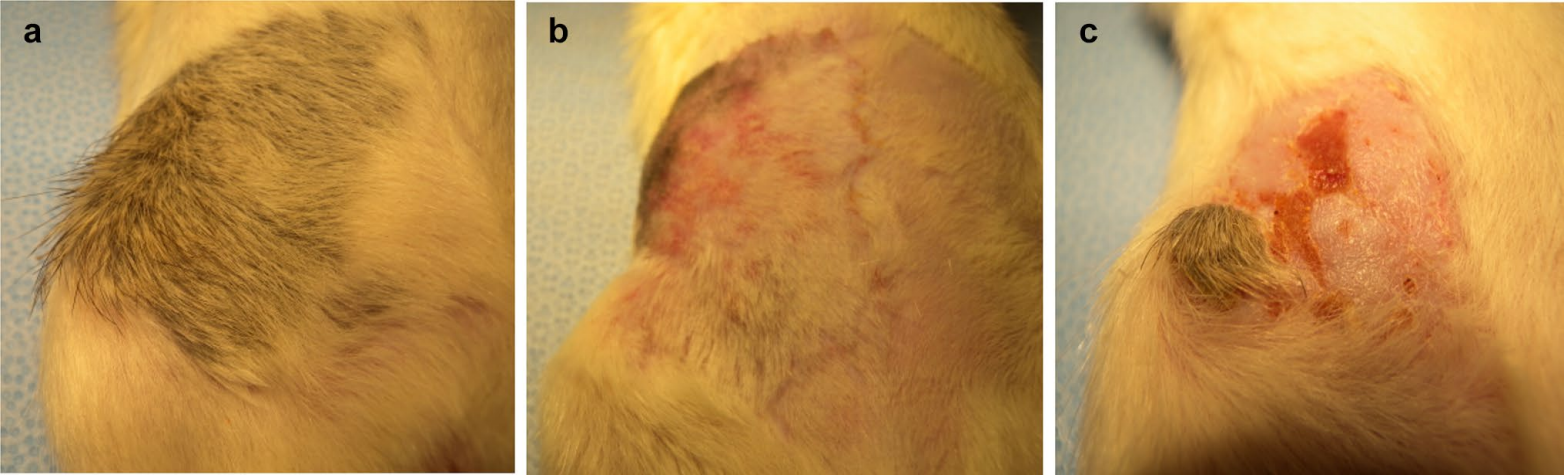
VCA (groin flap) recipient supported DRCC (2–4 × 10^6^ cells) therapy and 7-day immunosuppression protocol of anti-αβTCR monoclonal antibody and CsA: **a** healthy VCA at day 21 post-transplant; **b** early signs of acute rejection at day 105 post-transplant; **c** rejection at 115 days post-transplant. The signs of acute rejection included hair loss, edema and epidermolysis (**b**) which progressed to ulceration, shrinkage and mummification of the graft (**c**)

**Fig. 3:**
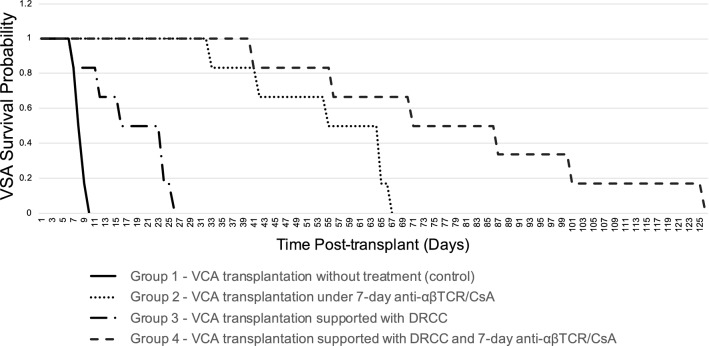
Kaplan–Meier survival curve of fully MHC-mismatched VCA transplanted from ACI (RT1^a^) donors to Lewis (RT1^l^) recipients

No complications following surgical procedure or DRCC injection were observed. No graft-vs.-host disease symptoms occurred following DRCC delivery. The transplanted VCA (groin flaps) were examined daily for signs of rejection (Fig. 2). The acute rejection occurred in all experimental groups. The average VCA survival time ranged from 8–125 days post-transplant (PT) and showed the following average survival times: Group 1 (untreated VCA recipient, control group) 8.5 ± 1 days PT, Group 2 (VCA recipients under 7-day anti-αβTCR/CsA IS protocol) 53.3 ± 13.6 days PT (*p* < 0.001 vs. Group 1), Group 3 (VCA recipients supported with DRCC therapy) 18 ± 7.5 days PT (*p* = 0.01 vs. Group 1 and *p* < 0.001 vs. Group 2), and Group 4 (VCA recipients supported with DRCC therapy and 7-day anti-αβTCR/CsA IS protocol) 79.3 ± 30.9 days PT. The VCA survivals in Group 4 were significantly prolonged in comparison to untreated VCA recipient controls (Group 1, *p* < 0.001), VCA recipients group supported with 7-day IS protocol (Group 2, *p* < 0.05) and VCA recipients supported with DRCC therapy (Group 3, *p* < 0.001). The Kaplan–Meier VCA survival curve is presented in Fig. 3.

### Application of Short-Term IS Protocol Facilitates the DRCC Engraftment and Development of Peripheral Blood Chimerism

**Fig. 4:**
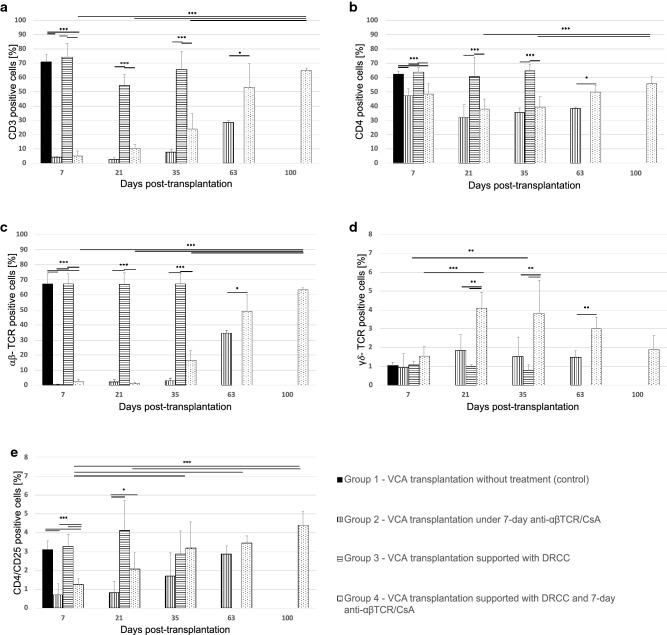
Comparison of the kinetics of different lymphocyte populations up to 100 days post-VCA transplantation. The levels of: **a** CD3, **b** CD4, **c** αβ-TCR, **d** γδ-TCR, and **e** CD4/CD25 lymphocytes were assessed in the peripheral blood of VCA recipients untreated or treated with supportive DRCC therapy and/or 7-day immunosuppression (IS) protocol of anti-αβTCR/CsA. The recipients of fully MHC-mismatched VCA received the following treatments: Group 1 (black)—VCA recipient without treatment, Group 2 (vertical pattern)—VCA recipient under 7-day IS protocol, Group 3 (horizontal pattern)—VCA recipients supported with DRCC therapy, and Group 4 (dot pattern)—VCA recipients supported with 7-day IS protocol and DRCC therapy. ***p < 0.001, **p < 0.01, *p < 0.05

The average percentage of T-cell population expressing CD3 in untreated (Group 1) and DRCC supported VCA recipients (Group 3) was 70.6 ± 5.4% and 74.4 ± 9.6%, respectively. Groups 2 and 4 which received the 7-day IS protocol showed a significant depletion of CD3 population (Group 2—4.2 ± 0.4%*** and Group 4—5.1 ± 3.5%***, ***p < 0.001 vs. Groups 1 and 3; Fig. 4a).

The decreased percentage of CD3-positive cells was maintained in Groups 2 and 4 on day 21 and 35 PT (day 21—2.7 ± 1.7%***, 10.4 ± 2.8%***, day 35—7.9 ± 4.6%***, 23.9 ± 10.8%***, respectively; ***p < 0.001 vs. Group 3). Further, the CD3 population recovery was detected at day 63 PT (Group 2—28.3 ± 5.8%* and Group 4—53.2 ± 16.5%, *p < 0.05 vs. Group 4). At day 100 PT the population of CD3-positive cells in Group 4 reached 64.5 ± 2.1%.

The application of IS protocol also decreased the average percentage of CD4 positive cells in Groups 2 and 4 between days 7 and 35 PT (day 7—47.1 ± 5.1%** and 48.5 ± 7.3%**, day 21—32.2 ± 8.7%** and 37.9 ± 6.8%**, day 35—35.8 ± 1.4%*** and 39.4 ± 6.9%***, respectively; **p < 0.01 vs. Groups 1 and 3; ***p < 0.001 vs. Group 3) compared to Group 1 and 3 (day 7—62.6 ± 2.1% and 64 ± 3%, respectively; Group 3: day 21—60.8 ± 13.2%, day 35—64.6 ± 4.7%). At day 63 PT, the CD4 population in Group 2 revealed 38.3 ± 1.4%, while in Group 4 reached 49.8 ± 4.2%* (*p < 0.05 vs. Group 2) and further increased to 55.9 ± 4.7% was detected at day 100 (Fig. 4b).

The depletion of cells expressing αβ-TCR at days 7 and 21 PT followed by recovery starting at day 35 in the IS treated VCA groups mirrored the pattern observed for CD3 cell population (day 7: Group 1—67.3 ± 6.7%, Group 2—0.5 ± 0.3%***, Group 3—67.2 ± 6.5%, Group 4—2.1 ± 1.8%***; ***p < 0.001 vs. Groups 1 and 3; day 21—Group 2—2.1 ± 1.4%***, Group 3—66.9 ± 7.7%, Group 4—1.2 ± 0.7%***; ***p < 0.001 vs. Group 3; day 35: Group 2—2.9 ± 1.7%***, Group 3—67.3 ± 6.5%, Group 4—16.6 ± 6.4%***; ***p < 0.001 vs. Group 3).

The αβ-TCR cell population continued to increase at day 63 PT (Group 2—34.7 ± 1.6%* and Group 4—48.9 ± 10.9%; *p < 0.05 vs. Group 4; Fig. 4c) reaching 63.4 ± 1.5% in Group 4 at 100 days PT.

There was no significant difference in the average percentage of cells expressing γδ-TCR between the VCA recipient group without treatment (Group 1: 1.06 ± 0.1%) and the VCA recipient group receiving IS protocol (Group 2: 0.92 ± 0.74%), DRCC therapy (Group 3: 1.1 ± 0.2%) or combined IS protocol and DRCC therapy (Group 4: 1.6 ± 0.5%) at day 7 PT (Fig. 4d). At day 21 the average percentage of γδ-TCR cells increased in Groups 2 and 4 to 1.85 ± 0.85% and 4.1 ± 0.9% ^#^,** (^#^*p* = 0.001 vs. day 7, **p < 0.01 vs. Groups 2 and 3), respectively. In comparison, Group 3 maintained the percentage of γδ-TCR cells at level of 0.98 ± 0.13% at day 21 and 0.78 ± 0.34% at day 35. Following increase at day 21, Group 2 also presented maintenance of γδ-TCR cells at level of 1.53 ± 1.02% and 1.48 ± 0.4% at days 35 and 63 PT. In comparison in Group 4, the population of γδ-TCR cells peaked at day 35 reaching 3.8 ± 1.8%** (**p < 0.01 vs. Groups 2 and 3) which was followed by decrease to 2.8 ± 0.94% at day 63 and 1.9 ± 0.8% at day 100 PT.

The assessment of the percentage of CD4/CD25 T regulatory cells at day 7 PT in Groups 2 and 4 supported by IS was significantly lower compared to Group 1 of untreated or Group 3 of DRCC only treated VCA recipients (Group 1—3.1 ± 0.5%, Group 2—0.7 ± 0.6%***, Group 3—3.3 ± 0.6%, and Group 4—1.3 ± 0.3%***; ***p < 0.001 vs. Groups 1 and 3, Fig. 4e). However, the average percentage of Treg population increased in all groups at day 21 PT (Group 2—0.8 ± 0.6%, Group 3—4.1 ± 1.6%*, Group 4—2.1 ± 0.8%*; *p < 0.05 vs. Group 2) and continued at days 35 and 63 PT in Group 2 (1.7 ± 1.2% at day 35 and 2.9 ± 0.4% at day 63) and Group 4 (3.2 ± 1.4% at day 35 and 3.5 ± 0.4% at day 63) with exception of Group 3 which showed a decrease to 2.9 ± 1.2% at day 35 PT; Fig. 4f. At day 100 PT, the population of Treg reached 4.4 ± 0.7% in Group 4.

### DRCC Therapy Induces Peripheral Blood Chimerism in the VCA Recipients

**Fig. 5:**
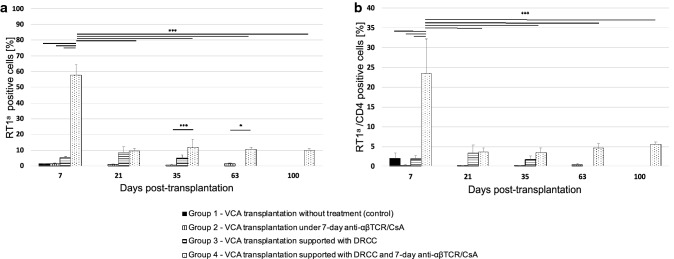
Comparison of the kinetics of (**a**) the total chimerism (RT1^a^) and (**b**) chimerism within the CD4 cell population (RT1^a^/CD4) detected in peripheral blood of the VCA recipients with or without the supportive therapy with DRCC and/or 7-day immunosuppressive protocol of anti-αβTCR/CsA. The recipients of fully MHC-mismatched VCA received the following treatments: Group 1 (black)—VCA recipient without treatment, Group 2 (vertical pattern)—VCA recipient under 7-day IS protocol, Group 3 (horizontal pattern)—VCA recipients supported with DRCC therapy, and Group 4 (check pattern)—VCA recipients supported with 7-day IS protocol and DRCC therapy. ***p < 0.001, *p < 0.05

During follow-up of the VCA transplants, the evaluation of total chimerism in control Group 1 which received no treatment and Group 2 supported only with anti-αβTCR/CsA protocol did not exceed 1.5% (Group 1: day 7—1.3 ± 0.2%; Group 2: day 7—1.4 ± 0.4%, day 21—0.9 ± 0.5%, day 35—0.83 ± 0.2% and day 63—1.35 ± 0.36%; Fig. 5a). The presence of ACI derived cells was detected in groups that received DRCC therapy. In Group 3 the average chimerism at day 7 was 5.1 ± 0.85%, at day 21—8.5 ± 3.8% and at day 35—5 ± 2.2%. At day 7, the total level of chimerism in Group 4 supported with IS and DRCC therapy reached 57.9 ± 6.2% ** was significantly higher compared to results in Groups 1, 2, and 3, **p < 0.01. Although the number of cells presenting RT1^a^ in Group 4 decreased during the follow-up period, the total chimerism level was maintained at the level of ~ 10% up to 100 days PT (day 21—9.4 ± 2%, *p* < 0.001 vs. day 7; day 35—12 ± 4.9%; day 63—10.5 ± 1.4%* and day 100—10 ± 1.5%; *p < 0.05 vs. Group 2; Fig. 5a).

The chimerism of CD4 cell population (RT1^a^/CD4) mirrored the pattern observed during the total chimerism analysis. At day 7 PT RT1^a^/CD4 chimerism level in Group 1 was at the level of 2 ± 1.2%, in Group 2—0.3 ± 0.2%, and in Group 3—1.9 ± 0.9%; Fig. 5b) were significantly lower compared to RT1^a^/CD4 chimerism detected in Group 4 (23.5 ± 8.7%***; ***p < 0.001 vs. Groups 1, 2, and 3). At day 21, the RT1^a^/CD4 chimerism in Group 4 significantly decreased to 3.6 ± 1%*** (***p < 0.001 vs. day 7) and was maintained at similar level at the following time points: 3.5 ± 1.2% at day 35, 4.7 ± 1%*** at day 63 and 5.5 ± 0.6%* at day 100 PT (***p < 0.001 vs. Group 2, *p < 0.05 vs. Group 2).

### Confirmation of Migratory Properties of DRCC In Vivo

**Fig. 6:**
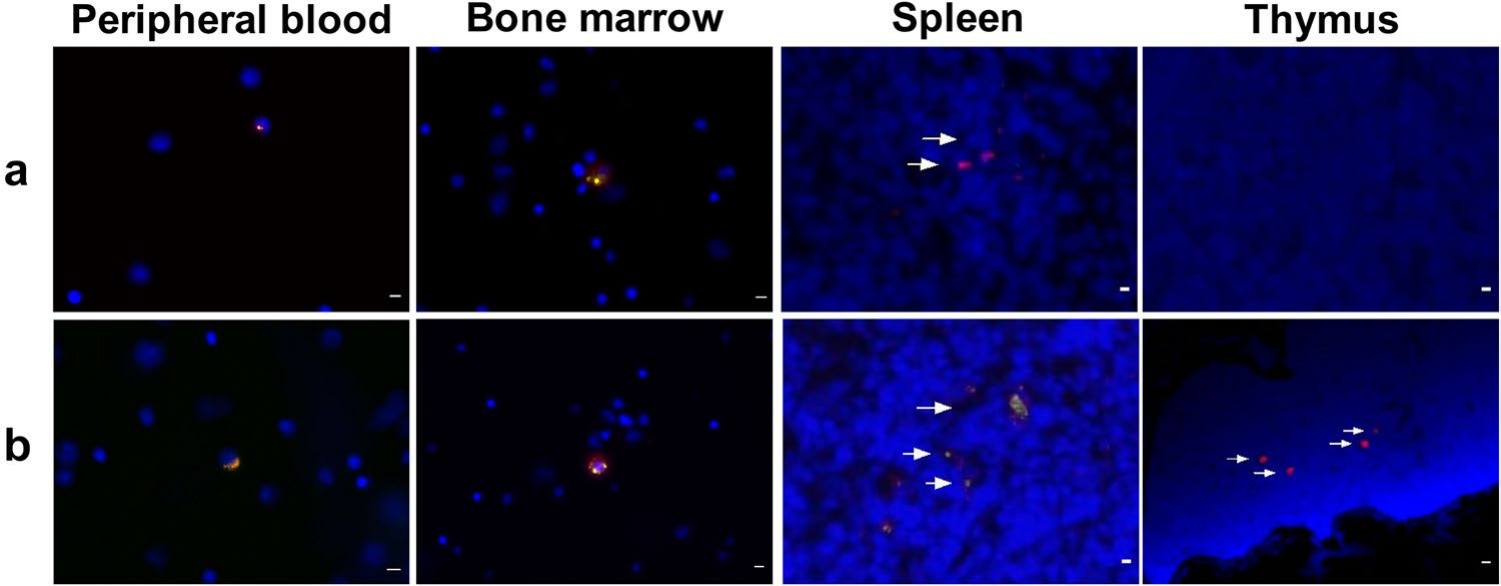
Representative fluorescence images confiming the long-term engraftment of DRCC transplanted as a supportive therapy to the recipients of VCA without immunosupression (day 25 post-transplant, (**a**) or receipients receiving combined DRCC and 7-day protocol of anti-αβTCR/CsA (day 100 post-transplant, (**b**). Double labeled PKH26/PKH67 DRCC were observed in the peripheral blood, BM harvested from the injected femoral bone, thymus and spleen of two VCA recipients at days which received suppotive therapy of DRCC and 7-day immunosupressive protocol of anti-αβTCR/CsA (Group 4), while in Group 3 where VCA recipients received only DRCC therapy, the double labeled PKH26/PKH67 DRCC were detected in the peripheral blood, BM, and spleen of one VCA recipient. For merge: Red: PKH26; Green: PKH67; Blue: DAPI (nuclei); Magnification × 20, scale bars 10 µm

The confocal microscopy and PCR analysis of peripheral blood, BM from the injected femur, thymus, spleen, and lymph node samples harvested at the endpoint in Groups 2, 3, and 4 assessed the presence of double labeled PKH26/PKH67 DRCC to determined the migratory properties of DRCC in vivo. No cells were detected in blood and BM samples of VCA recipients supported only with IS protocol (Group 2). The DRCC were found in the peripheral blood and BM of one of VCA recipients supported with DRCC therapy (Group 3) and two VCA recipients supported with IS protocol in combination with DRCC (Group 4); Fig. 6.

Similarly, the analysis of the lymphoid organs using fluorescent microscopy indicated the presence of DRCC in thymus and spleen of two VCA recipients in Group 4 (Fig. 6). In addition, the DRCC were also observed in the spleen of one VCA recipient in Group 3, however, no cells were present in the thymus. No cells were detected in thymus and spleen of VCA recipients in the control Group 2 as well as in the samples of lymph nodes in any of the experimental groups.

**Fig. 7:**
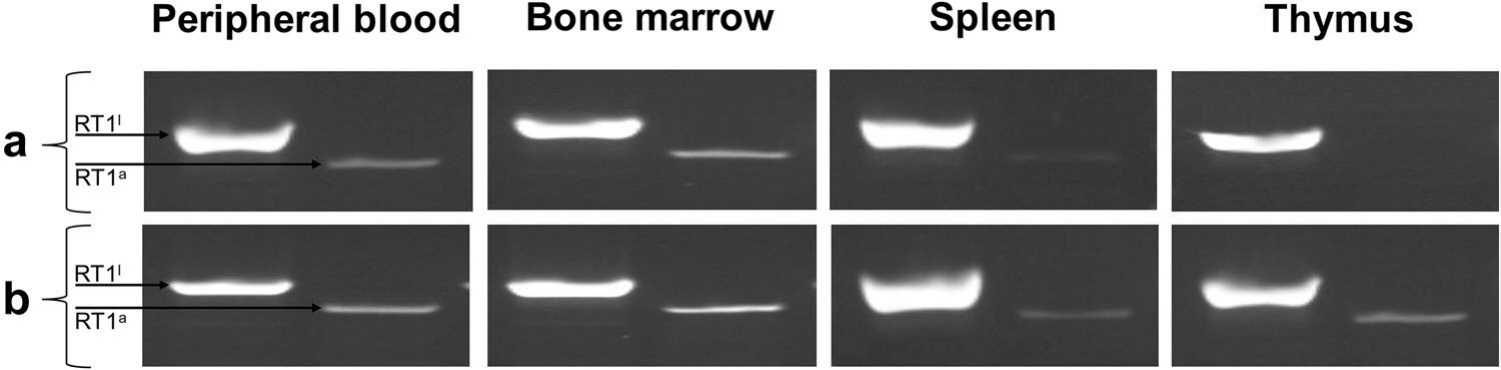
Representative polymerase chain reaction (PCR) results confirming the presence of donor-derived cells in the VCA (groin flap) recipients receiving DRCC without immunosuppression (Group 3, A) or VCA recipients receiving DRCC combined with the 7-day IS protocol of anti-αβ-TCR/CsA (Group 4, B). Samples of peripheral blood, BM, spleen, and thymus of VCA recipients from Groups 2, 3, and 4 collected at the endpoint of experiments (VCA rejection) were analyzed using PCR detecting MHC class I sequences specific for ACI (RT1a; 388 bp) and Lewis (RT1l; 448 bp) rats. The analysis confirmed the presence of donor-derived cells in the peripheral blood, BM, and spleen of VCA recipients in Group 3 and peripheral blood, BM, spleen, and thymus of VCA recipients in Group 4

The findings of PCR analysis detecting the presence of MHC class I sequences specific for ACI (RT1^a^) donors in the VCA recipients supported the results of flow cytometry detecting peripheral blood chimerism and microscopy detecting the long-term mainenance of DRCC in vivo (Fig. 7).

## Discussion

The concept of autologous and allogeneic cell-based therapies has been gaining momentum in recent years in almost all branches of medicine. In the field of transplantation, the encouraging results of experimental and clinical BMT studies (Cowan et al. 2012; Deng et al. 2004; Foster et al. 1998; Garcia-Morales et al. 1998; Leventhal et al. 2012; Siemionow et al. 2002b, 2008) propelled interest in development of novel targeted cell therapies for tolerance induction (Hivelin et al. 2016; Hutchinson et al. 2008; Kaufman et al. 1994; Scandling et al. 2018; Singh et al. 2018).

We introduced a novel DRCC which we have created in vivo (Hivelin et al. 2016) and ex vivo via PEG-mediated cell fusion (Cwykiel et al. 2021). The proposed approach aimed to create cell therapy supporting tolerance induction in solid organ and VCA recipients offering mixed donor and recipient phenotype, decreased immunogenicity and improved engraftment and chimerism development without myeloablative conditioning of transplant recipient. In our previous in vitro study (Cwykiel et al. 2021), we established the protocol to create DRCC ex vivo and confirmed the decreased immunogenicity of DRCC as well as pro-tolerogenic profile of cultured DRCC presented by secretion of IL-10 and TGFβ1. Moreover, DRCC demonstrated proliferation and differentiation capabilities comparable to BMC controls.

These promising results encouraged us to assess immunomodulatory properties of DRCC in vivo as a supportive therapy for VCA recipients. DRCC testing was performed in a rat groin flap transplantation model (Demir et al. 2005). The DRCC were delivered using our well-established protocol of intraosseous injection (Klimczak et al. 2007). We have previously confirmed that intraosseous route of cell delivery improves engraftment of injected cells by reduction of cell sequestration from the blood stream, and, therefore, is more effective for long-term chimerism induction compared to the intravenous injection (Klimczak et al. 2007; Siemionow et al. 2005b). In addition, intraosseous cells delivery is safer compared to intravenous route since it prevents cells from lodging in the lungs. Recent reports on intraosseous delivery of cord-blood cells and mesenchymal stem cell in the clinical trials confirm our findings of better cell engraftment and safety of the procedure (Bonifazi et al. 2019; Döring et al. 2020; Frassoni et al. 2008; Goto et al. 2018; Lee et al. 2013; Marktel et al. 2019). The DRCC therapy was tested with and without a 7-day non-myeloablative IS protocol of anti-αβTCR/CsA. The rationale for selection and duration of the IS protocol was based on our previous studies assessing the 7-day IS protocol in rat limb, face and groin flap VCA models (Demir et al. 2005; Hivelin et al. 2016; Ozer et al. 2004; Siemionow and Klimczak 2013; Siemionow et al. 2002b, 2003), which confirmed its efficacy in extending VCA survival and supporting chimerism induction.

In this study, application of DRCC therapy significantly extended VCA survival compared to the untreated controls, specifically when supported with 7-day IS protocol where the longest allograft survival (125 days) was observed. In contrast, the longest survival of VCA under IS protocol (Group 2) reached 67 days which was in line with previously published groin flap survival data under the same IS protocol (Demir et al. 2005). We also observed a significant extension of allograft survival in the study testing in vivo created DRCC under the same IS protocol in a face allotransplantation model (Hivelin et al. 2016) which also confirms the immunomodulatory effect and pro-tolerogenic properties of DRCC therapy. Interestingly, in vascularized BMT model 100% allograft survival was achieved up to 100 days PT under 7-day anti-αβTCR/CsA IS protocol (Siemionow et al. 2008) and transplantation of VCA combined with vascularized bone component significantly extended allograft survival (125 days) compared to 50% reduction of survival in VCA transplant without bone component despite application of the same IS protocol (Ozmen et al. 2006a). The higher survival rates observed in the VCA models containing vascularized bone component validate previous reports on the immunomodulatory role of BMCs supporting development of donor-derived chimerism and tolerance in VCA transplants (Siemionow and Klimczak 2013; Siemionow et al. 2003, 2008).

We hypothesize that application of DRCC therapy in VCA models containing vascularized bone component may accelerate development of a stable long-term macrochimerism. The synergistic effect of regulatory cell populations which are present within the BMC and tolerogenic properties of DRCC will lead to improved VCA survival and tolerance induction in the transplant recipients.

As confirmed by clinical transplant studies, VCA transplants, such as hand or arm, do not contain sufficient amount of red marrow to promote HSC engraftment and establish long-term chimerism (Szajerka et al. 2011). Thus, from the clinical perspective, transplanting vascularized bone in the context of VCA as a tolerance inducing strategy is challenging. Intraosseous delivery of higher dose or multiple dosages of DRCC therapy provides similar or even superior support of VCA transplants without the need for immunosuppression and additional surgical procedure.

Our IS protocol used in this study confirmed previously reported (Ozer et al. 2004; Siemionow et al. 2003) significant depletion of the CD3 and αβ-TCR expressing lymphocyte populations at early post-transplant period and start of the recovery of depleted cells around day 21 PT. The comparison between groups with and without application of anti-αβTCR/CsA IS protocol, confirmed selective depletion and preservation of the γδ T cell population (Siemionow and Klimczak 2009) which is known to fight infection, development of tumors and have modulates activity of the effector cells involved in local immune response in tissues, such as skin (Fujihashi et al. 1999; Locke et al. 2006; Nanno et al. 2007).

The application of 7-day IS protocol resulted in significant > 40% decrease of the CD4 population at day 21 post-transplant in groups supported with DRCC therapy when compared to the untreated controls. It has been shown in vitro and in vivo that CsA halts development of CD4 expressing cells and interferes with CD4 mediated responses (Fischer et al. 1991). Analogously, application of αβ-TCR/CsA protocol significantly reduced the number of Tregs in VCA recipients compared to the control groups without IS. However, the application of DRCC therapy resulted in more efficient recovery of Treg cell population leading to “rescue” and higher numbers of Tregs over time, which was not observed in VCA recipients receiving IS without DRCC supportive therapy. Replacing the CsA with either tacrolimus or rapamycin in the IS protocol could improve the efficacy of the DRCC therapy by increasing the Treg population, thus supporting development of tolerogenic effect via peripheral tolerance mechanism (Stallone et al. 2016).

We have observed maintenance of the long-term macrochimerism in the peripheral blood of VCA recipients supported with DRCC therapy, whereas microchimerism was observed in VCA treated with IS protocol only which correlated with significantly reduced allograft survival.

Our study confirmed that DRCC are capable to engraft with or without application of a short-term IS protocol; however, under the 7-day IS, the total chimerism levels were 10-times higher in VCA recipients receiving combined IS and DRCC therapy compared to the recipients receiving only IS protocol. Moreover, this significantly higher chimerism level was maintained over the entire follow-up period only in the combined DRCC and IS therapy group, whereas gradual chimerism decline was observed under other treatment protocols. These results are comparable with chimerism levels observed in VCA transplants supported with BMT (Klimczak et al. 2007).

In addition, chimerism kinetics for CD4 cell population (RT1^a^/CD4 were maintained at 5% level up to the experimental endpoint only in VCA recipients treated with combined DRCC and IS protocol, which is in line with previously published study testing 7-day αβ-TCR/CsA protocol in VCA models (Demir et al. 2005; Siemionow et al. 2005b).

We have confirmed that the presence of vascularized bone component of the VCA transplant provides a constant source of the donor-derived cells leading to the development of stable chimerism induction at high levels (20–25%) up to 100 days post-transplant in the MHC-mismatched limb transplants supported with 7-day αβ-TCR/CsA which was associated with tolerance induction and allograft survival over 350 days post-transplant (Siemionow et al. 2003). However, in the clinical scenario transplantation of VCA including substantial bone component to induce tolerance is technically demanding and not always possible thus, different experimental models of VBMT were proposed as an alternative methods for tolerance induction (Gordon et al. 2007; Klimczak et al. 2006; Siemionow et al. 2008). Our study assessing development of chimerism in VBMT model under 7 day anti-αβTCR/CsA showed a gradual chimerism decline reaching 1% at 100 days PT which was associated with the gradual development of fibrosis within the donor’s bone (Klimczak et al. 2006; Siemionow and Klimczak 2013). Thus, intraosseous delivery of cell therapy to the BM niche as performed in the current study may overcome this hurdle since it will lead to more effective engraftment of cells allowing for maintenance of the long-term chimerism.

We have also confirmed by confocal and fluorescent microscopy and PCR, long-term maintenance of chimerism which correlated with the presence of DRCC in the BM compartment, as well as thymus and spleen at the study endpoint confirming migratory capabilities of DRCC. These findings may provide foundation for the possible mechanism of action of DRCC in vivo, showing immunomodulatory effects, thus altering the T cell repertoire by presenting mixed antigens on DRCC surface and/or acting as cells stimulating the regulatory cells via cytokine signaling. Studies in solid organs, VCA and BMT reported migration of donor-derived cells to the recipient’s lymphoid tissues and organs as well as lungs, skin and liver and confirmed their role in tolerance induction process (Khan et al. 1996; Ozmen et al. 2006b; Siemionow et al. 2006; Zor et al. 2020). Both thymus and spleen are considered as immune cell rich organs participating in innate and adaptive immune response (Dor et al. 2003; Gagliani et al. 2013). Studies testing effect of thymectomy on transplantation tolerance confirmed the significance of thymus in tolerance induction mechanisms (Vagefi et al. 2004) and correlation of the BMC engraftment and thymic chimerism with central tolerance induction (Horner et al. 2006). Spleen, the secondary lymphoid organ, has been also associated with immunomodulation contributing to alloantigen tolerance induction by harboring the myeloid derived suppressor cells (Wang et al. 2013) and MSC (Krampera et al. 2007; Sakata et al. 2018), as well as creating permissive environment to induce regulatory cells, such as Treg (Getts et al. 2011; Masli et al. 2002). These studies confirming immunomodulatory role of thymus and spleen support our findings of long-term chimerism maintenance which correlated with the presence of DRCC in spleen and thymus confirmed by immunofluorescence imaging.

The groin flap VCA model containing highly immunogenic skin component was selected to challenge the in vitro confirmed immunomodulatory properties of DRCC therapy. Although we did not achieve tolerance, our study was in line with previous reports confirming that total chimerism level below 20% was associated with acute rejection of the allograft (Dubernard et al. 2007; Foster et al. 1998; Petruzzo et al. 2003; Siemionow and Klimczak 2013; Siemionow et al. 2005a); However, our study highlighted the role of DRCC in establishing long-term chimerism where the possible mechanism of action of DRCC would be based on the recipient’s immunomodulation via central (confirmed the presence of DRCC in the thymus) or peripheral tolerance (confirmed the presence of DRCC in the spleen). The limitations of this study include the lack of assessment of the direct and indirect interactions between DRCC and VCA recipient’s immune cells in vivo; however, it provides basis for future in vivo exploration of the mechanism of DRCC action focusing on their involvement in the immunoregulatory processes to increase the efficacy of DRCC for the potential therapeutic application in the clinical scenario of solid organs, VCA, and BM transplantation.

Since the clinical BM transplantation and living donor solid organ transplantation, such as kidney or liver, are elective procedures due to donor availability, the DRCC therapy can be prepared in advance and delivered at the day of transplantation. In clinical cases of VCA transplantation access to the large volume of the donor BM will allow to create sufficient number of cells via multiple cell fusions which will be sufficient for the first peri-transplant dose of DRCC. Propagation of DRCC after transplant will allow for delivery of additional doses based on the assessment of donor chimerism. Moreover, DRCC can be cryopreserved after culture and administrated at long-term follow-up to support VCA survival in case of signs of chronic graft rejection. Thus, DRCC represent novel therapeutic approach for the maintenance of chimerism and extension of VCA survival.

## Conclusions

This proof of concept study confirmed in vivo the immunomodulatory potential of DRCC therapy and its role in induction and maintenance of long-term chimerism which correlated with significant extension of VCA survival when combined with short 7-day αβ-TCR/CsA IS protocol. Thus, DRCC represents a novel promising approach for clinically feasible customized donor-recipient cell-based therapy with immunomodulatory properties supporting development of chimerism in solid organs and VCA transplants.

## Data Availability

All data generated in this study are included in the manuscript and are available for presentation upon request.
